# Analysis of hub genes temporal dynamics in cardiac ischemia/reperfusion injury: a bioinformatics and experimental study

**DOI:** 10.3389/fphys.2025.1668586

**Published:** 2025-11-19

**Authors:** Yu Zuo, Si-Yuan Yang, Xing-Kai Qian, Kun Liu

**Affiliations:** 1 Cardiac Surgery Division, The Affiliated Hospital of Guizhou Medical University, Guiyang, Guizhou, China; 2 Guizhou Medical University’s Translational Medicine Research Institute, Guiyang, Guizhou, China

**Keywords:** myocardial ischemia/reperfusion injury, bioinformatics analysis, gene expression omnibus, differential expression genes, hub genes, enrichment analysis

## Abstract

**Introduction:**

Myocardial ischemia/reperfusion injury (MIRI) remains a major global health issue with rising morbidity and mortality. Blood flow restoration can worsen damage to oxygen-deprived cardiac tissue. This research aimed to identify hub genes linked to MIRI across various reperfusion stages, offering potential diagnostic indicators and therapeutic targets.

**Methods:**

Differentially expressed genes (DEGs) at 6, 24, and 72 h post-reperfusion based on the GSE160516 dataset via GEO2R were identified, and these genes were further analyzed using the DAVID database and online platforms for Gene Ontology Kyoto Encyclopedia of Genes and Genomes (KEGG) and Disease Ontology (DO) enrichment. Protein–protein interaction networks were constructed using STRING and visualized with Cytoscape, and the Degree algorithm within the CytoHubba plug-in identified the top ten hub genes at each time point. Additional analyses included miRNA-hub gene prediction, transcription factor-hub gene regulation, and drug-hub gene interactions via miRDB, TRRUST and DGIdb platforms. Receiver operating characteristic (ROC) curve analysis and immune cell infiltration assessment were performed using online analytical tools and CIBERSORT. Finally, an oxygen–glucose deprivation/reperfusion (OGD/R) model in HL-1 mouse cardiomyocytes and external datasets validated the expression of hub genes.

**Results:**

Totally, 341, 399 and 431 DEGs at 6, 24, and 72 h post-reperfusion were screened, enriched in functions, such as inflammatory pathways, cytokine-receptor interactions, and immune or inflammatory-related diseases. Degree algorithm analysis identified the top ten hub genes (Il1b, Cxcl2, Cd44, etc.) at 6h, (Il1b, Il6, Fn1, etc.), at 24 h and (Fcgr3, Ccl2, Cd68, etc.) at 72 h. Most hub genes showed notable interactions with specific microRNAs (mmu-let-7c-1-3p, mmu-miR-466L-3p), the transcription factor Nfkb1, and drugs with the highest drug-gene interaction scores (EMAPTICAP PEGOL, CARLUMAB, Compound 27 [PMID: 19854648]). ROC curve analyses demonstrated high diagnostic potential (AUC 0.889–1.000; sensitivity 100%; specificity 66.67%–100%) and immune infiltration analysis indicated increased resting dendritic cells and decreased neutrophils. Validation in HL-1 OGD/R models and external datasets confirmed elevated mRNA expression of Serpine1, Ccl2, Il6, and Fn1 at various reperfusion stages.

**Conclusion:**

Analysis across multiple datasets and HL-1 cell experimental models revealed four significantly altered genes—Serpine1, Ccl2, Il6 and Fn1—indicating their potential targets for MIRI.

## Introduction

Coronary heart disease (CHD) remains the leading cause of death and disability worldwide. By 2020, it was estimated that approximately 244.1 million people globally were living with ischemic heart disease (Feigin et al., 2022). A major contributor to CHD is myocardial ischemia-reperfusion injury (MIRI), which occurs when blood flow is restored to the heart tissue after a period of ischemia. Although reperfusion therapy can reduce ischemic damage and limit the extent of heart attacks, the restoration of blood flow paradoxically exacerbates tissue injury, a phenomenon known as ischemia-reperfusion (I/R) injury (Wang et al., 2021; Wu et al., 2021; Zheng et al., 2021).

Recent advancements in transcriptome sequencing have positioned bioinformatics as a crucial tool for the discovery of novel diagnostic and therapeutic biomarkers. The integration of molecular biology and bioinformatics has enabled large-scale analyses of publicly available datasets, facilitating the study of diseases at a molecular level. These data-driven approaches create significant opportunities to enhance our understanding the occurrence and development of the disease (Li et al., 2018). In the context of MIRI, recent bioinformatic studies have identified several abnormally expressed hub genes that play central roles in biological networks, such as SOCS3, CXCL1, IL1B, and MMP-9 (Wu et al., 2024), as well as HIF-1α, BNIP3, and LC3 (Chen et al., 2024). Additionally, dysregulated microRNAs (miRNAs), including mmu-let-7f-1-3p, miR-706, and miR-466j (Hu et al., 2023), have been implicated in the post-ischemic reperfusion response. These altered genes and miRNAs are believed to be involved in molecular pathways regulating angiogenesis (Ye et al., 2022), mitophagy (Chen et al., 2025), and ferroptosis (Ravingerová et al., 2020), all of which play critical roles in MIRI. Despite these advances, research on gene expression changes in MIRI at various reperfusion time points remains limited. To address this gap, the oxygen-glucose deprivation/reperfusion (OGD/R) model using HL-1 cell lines—a mouse cardiac muscle cell line—offers a promising system for revealing gene expression profiles changes involved in this process. This approach will enhance our understanding of MIRI and offer new insights into myocardial protection.

Previous studies have employed microarray analysis to explore molecular changes in myocardial ischemia-reperfusion injury (Wang et al., 2022; Chen et al., 2024). While differentially expressed genes (DEGs) and hub genes associated with MIRI have been identified, no comprehensive study has examined the temporal dynamics of their expression levels, nor the networks involving miRNAs, transcription factors, and drug interactions. To fill this gap, our study analyzed the publicly available microarray dataset GSE160516, systematically comparing gene expression profiles between sham and MIRI at three critical reperfusion time points. This analysis aimed to identify key DEGs and hub genes, followed by functional and interaction analyses that provide novel insights into the biological processes underlying myocardial injury and repair during reperfusion. Collectively, our findings advance the understanding of MIRI’s molecular underpinnings and may inform future therapeutic targets.

## Methods

### Datasets downloading and preprocessing

The GSE160516 dataset, sourced from the GEO repository (http://www.ncbi.nlm.nih.gov/geo/), encompasses gene expression data from both healthy and MIRI tissue samples. The data was generated using a GPL23038 microarray called the [Clariom_S_Mouse] Affytrix Clariom S Assay (Affytrix, Santa Clara, CA), using Pico Assay technology and converted into unified genetic symbols. After blocking the left anterior descending coronary artery (LDA), blood flow returned for 6 h, 24 h, and 72 h, respectively. Data included 4 samples from the control group (GSE4874400-GSE4874403) from sham surgery, and 4 samples from each group of the following: IR-6h group (GSE4874404-GSE4874407), IR-24 h group (GSE4874408-GSE4874411), and IR-72 h group (GSE4874412-GSE4874415).

### Data processing of DEGs

We used the GEO2R tool to find DEGs after blood flow restoration in the sham group and the MIRI group respectively (Barrett et al., 2012). The criteria for gene selection were: the adjusted P value was less than 0.05 and the absolute log fold change was greater than or equal to 2, and then these genes were screened out at each time point for further analysis. We used the online Venn map tool to find out the DEGs that were common to different ischemia-reperfusion (IR) groups and see which genes were common to everyone. This approach can help us understand how these genes may affect reperfusion injury. We also used unsupervised principal component analysis (PCA) to identify the main characteristics from three time points after blood flow resumed. Heat maps and volcanic maps were drawn using the bioinformatics website (https://www.bioinformations.com.cn) to display DEGs. These maps allow us to see gene expression and statistical significance.

### Gene ontology of differentially expressed genes

To understand what role those genes with altered expression levels (DEGs) play in different functional areas such as biological activity (BP), intracellular location (CC), and biochemical activity (MF), we conducted detailed enrichment analysis. We used the famous Gene Ontology (GO) (Ashburner et al., 2000); Kyoto Encyclopedia of Genes and Genomes (KEGG) (Altermann and Klaenhammer, 2005) databases, and also used the DAVID web tool (https://david.ncifcrf.gov/) to simplify functional classification. Statistical significance uses a strict adjusted p-value criterion (<0.05) to ensure that only truly important pathways and functions are studied. To further investigate the functional implications of the differentially expressed genes (DEGs), we conducted disease ontology (DO) (Schriml et al., 2022) enrichment analysis using OmicShares online platform (http://www.omicshare.com/tools). This approach allowed us to systematically pinpoint diseases linked to the DEGs, providing a robust foundation for interpreting their biological relevance. By leveraging this comprehensive analytical tool, we ensured the validity and reliability of our findings regarding the functional impact of these genes.

### Determining the protein-protein interactions (PPIs) network and hub genes

To examine protein-protein interactions (PPIs), we leveraged the STRING database (https://www.string-db.org/) (Szklarczyk et al., 2019), setting an interaction score threshold of >0.4 to identify meaningful associations. The resulting PPI networks were then mapped and analyzed using Cytoscape 3.9.1 (Kohl, 2011). Key Hub genes were pinpointed via the Cytohubba plugin, with the top 10 highest-degree nodes designated as central players in each time-specific network. Additionally, we employed the MCODE plugin to identify densely interconnected modules, applying default parameters—degree cutoff: 2, node score cutoff: 0.2, K-core: 2, and maximum depth: 100—to isolate the most significant molecular clusters and seed genes.

### miRNA/transcriptional factor-hub gene interplay analysis

The miRNA Prediction Database (miRDB, https://mirdb.org/) provides the most comprehensive miRNA-mRNA interaction network available to date (Chen and Wang, 2020). Using this resource, we identified miRNAs that target key hub genes at multiple reperfusion intervals. We then prioritized the miRNAs that regulate the largest number of hub genes and mapped overlapping miRNAs across different ischemia-reperfusion groups using Venn diagrams. For transcriptional network analysis, we employed TRRUST (https://www.grnpedia.org/trrust/), a robust platform for studying human and mouse transcriptional regulation (Han et al., 2018). This database catalogs 8,444 transcription factor (TF)-target interactions involving 800 human transcription factors, offering critical insights into multi-gene regulatory mechanisms. Using TRRUST, we predicted transcription factors influencing hub genes across different reperfusion phases, identified those with the broadest regulatory effects across the three reperfusion stages, and visualized shared transcription factors among ischemia-reperfusion groups using Venn diagrams. Finally, we used Cytoscape software (Shannon et al., 2003) to generate integrated regulatory networks that incorporate both miRNA-hub gene and TF-hub gene interactions.

### Gene-drug interaction analysis

The Drug–Gene Interaction Database (DGIdb, www.dgidb.org) is an online resource that compiles information on drug–gene interactions and druggable genes derived from publications, databases, and other web-based sources. It serves as a tool for identifying drugs that interact with these genes (Freshour et al., 2021). In this study, we used the DGIdb to predict potential drugs and small molecule compounds that interact with hub genes at various reperfusion time points. We focused on screening candidate drugs with the highest interaction scores across three distinct reperfusion time points. A Venn diagram was constructed to identify drugs and small molecule compounds that co-regulate the three time points. Additionally, we employed Cytoscape software (Shannon et al., 2003) to map the interaction network between the identified drugs and hub genes.

### ROC curve for the diagnostic potential of hub genes

We obtained the GSE160516 along with three external gene expression datasets (GSE193997, GSE58486, and GSE61592) representing different reperfusion time points. To assess the diagnostic performance of hub genes across these time points, we constructed receiver operating characteristic (ROC) curves using the OmicShare Online Analysis Tool (http://www.omicshare.com/tools). The diagnostic metrics, including the area under the curve (AUC), specificity, sensitivity, and cutoff values for the hub genes, were analyzed using GraphPad Prism 10.0 software (GraphPad Software, Inc., San Diego, CA, United States). The AUC value was used to assess the diagnostic accuracy, as it reflects both the true positive rate and the true negative rate of each gene. An AUC approaching 1 indicates excellent diagnostic performance, highlighting the potential clinical applicability of the genes in medical practice.

### Immune cell infiltration analysis in GSE160516

To delve into the intricacies of gene expression, the CIBERSORT algorithm was employed to scrutinize the normalized data. This high-tech tool allowed for the precise measurement of 22 diverse immune cell populations within each sample. Before the analysis could proceed, the “limma” package was utilized to bring the dataset to a standard level. Following normalization, the data were then subjected to CIBERSORT, a process that, using the R programming language, translated the data into the specific profiles of the 22 immune cell varieties. To polish the findings further, a Perl-based filtering technique was applied, setting a stringent threshold at P < 0.05, available through https://www.perl.org. This meticulous approach produced a detailed infiltration matrix of immune cells infiltration matrix. For visualization, correlation analyses and the 22 immune cell types were plotted using the R package corrplot, while differences in immune infiltration between the MIRI and sham-operated groups across various reperfusion intervals were illustrated with vioplot. These methods provided detailed insights into the immune landscape of the samples.

### External datasets validation

To continue our research, we obtained several external datasets directly from the GEO database-GSE193997, GSE58486 and GSE61592. First, GSE193997 is sequencing data on cardiac RNA, including samples from three mice that had 6-h myocardial ischemia-reperfusion injury (MIRI-6h) and three normal mice as controls. GSE58486 is data that measures mRNA in heart tissue. There are data on three mice that experienced 24-h (MIRI-24 h), as well as a group of control mice. This dataset uses the GPL18802 platform. Finally, GSE61592 is microarray data to study late cardiac responses, including three mice that had 72 h (MIRI-72 h), and four control mice. It is based on the GPL6887 platform, so that data can be compared in detail at all points in time.

### HL-1 cell culture and oxygen-glucose deprivation/reperfusion injury model

HL-1 cell lines incubated in DMEM (#11965092; Gibco, United States) medium supplemented with 10% FBS (#A5256701; Gibco, United States) and placed in an incubator at 37 °C and 5% CO_2_. To simulate hypoxia, HL-1 cells were exposed to an anoxic chamber containing a mixture of 95% N_2_ and 5% CO_2_ for 6 hours. After the hypoxia experiment, the cells were returned to normal oxygen environment for recovery, which ranged from 6 h to 72 h. For comparison, we also raised a group of HL-1 cells as a control group and kept them under normal oxygen conditions.

### Cell viability assay

To gauge the viability of the HL-1 cells, the Cell Counting Kit-8 (CCK-8; #K1018, APExBIO, out of the U.S.) was deployed. The cells were sown in 96-well dishes at a ratio of 2,000 cells per container. The cells underwent oxygen-glucose deprivation/reperfusion experiments the following day. After different reperfusion time points, each well was replaced with 100 μL serum-free medium containing 10 μL CCK-8 reagent. The plates were then incubated at a cozy 37 C for a spell of 4 hours. Following the incubation period, the absorbance at 450 nm was taken with the aid of a nifty microplate scanner (Fully Automatic Microplate Reader; #WD-2102B, Beijing Liuyi Biotechnology Co., Ltd.). The data on cell vitality were aligned with those of the comparative control group.

### Quantitative real-time polymerase chain reaction

RNA was extracted from HL-1 cells using TRIzol reagent (Invitrogen, United States) following the manufacturer’s protocol. Purified total RNA (1 μg) was reverse transcribed into complementary DNA (cDNA) using the PrimeScript RT Kit (Takara, Dalian). Gene expression was quantified using a Bio-Rad (United States) real-time PCR machine, with SYBR Green (Takara) as the fluorescent dye. Thermocycling conditions were established according to the manufacturer’s recommendations. The reaction commenced with an initial denaturation at 95 °C for 30 s, followed by 40 amplification cycles consisting of denaturation at 95 °C for 5 s and annealing/extension at 60 °C for 30 s mRNA expression levels were calculated using the 2^(-ΔCt)^ method. To assess relative changes in gene expression, the 2^(-ΔΔCt)^ method was applied after normalizing to the control group. β-actin served as the endogenous control to normalize expression levels for each target gene. Primer sequences used for PCR are listed in Table 1.

**TABLE 1:** Primers for real-time polymerase chain reaction.

Gene	Forward primer	Reverse primer
IL-6	TAGTCCTTCCTACCCCAATTTCC	TTGGTCCTTAGCCACTCCTTC
CCL-2	TTAAAAACCTGGATCGGAACCAA	GCATTAGCTTCAGATTTACGGGT
ITGAM	CCATGACCTTCCAAGAGAATGC	ACCGGCTTGTGCTGTAGTC
IL-1β	GCAACTGTTCCTGAACTCAACT	ATCTTTTGGGGTCCGTCAACT
CXCL2	GGAAGCCTGGATCGTACCTG	TGAAAGCCATCCGACTGCAT
CD44	TCGATTTGAATGTAACCTGCCG	CAGTCCGGGAGATACTGTAGC
CXCL10	CCAAGTGCTGCCGTCATTTTC	GGCTCGCAGGGATGATTTCAA
SERPINE1	TCTGGGAAAGGGTTCACTTTACC	GACACGCCATAGGGAGAGAAG
CXCL1	ACTGCACCCAAACCGAAGTC	TGGGGACACCTTTTAGCATCTT
PTGS2	TTCAACACACTCTATCACTGGC	AGAAGCGTTTGCGGTACTCAT
FN1	ATGTGGACCCCTCCTGATAGT	GCCCAGTGATTTCAGCAAAGG
PTPRC	GTTTTCGCTACATGACTGCACA	AGGTTGTCCAACTGACATCTTTC
ITGB2	CAGGAATGCACCAAGTACAAAGT	GTCACAGCGCAAGGAGTCA
FCGR3	AATGCACACTCTGGAAGCCAA	CACTCTGCCTGTCTGCAAAAG
TNF	CAGGCGGTGCCTATGTCTC	CGATCACCCCGAAGTTCAGTAG
TYROBP	CCCAAGATGCGACTGTTCTTC	GTCCCTTGACCTCGGGAGA
CD68	TGTCTGATCTTGCTAGGACCG	GAGAGTAACGGCCTTTTTGTGA
CSF1R	TGTCATCGAGCCTAGTGGC	GGTCCAAGGTCCAGTAGGG
ADGRE1	TGTCTGAAGATTCTCAAAACATGGA	TGGAACACCACAAGAAAGTGC
ACTB	GTGACGTTGACATCCGTAAAGA	GCCGGACTCATCGTACTCC

### Statistical analysis

The data presented here are in terms of the average and standard error of the mean. The analysis was performed with the help of IBM SPSS Statistics, version 21.0. To compare the two groups, a Student’s t-test was applied. Variations among the components were evaluated through a one-way ANOVA, with further examination via Tukey’s follow-up test. We deemed any results with a p-value below 0.05 as statistically meaningful.

## Results

### Identification of DEGs

**FIGURE 1:**
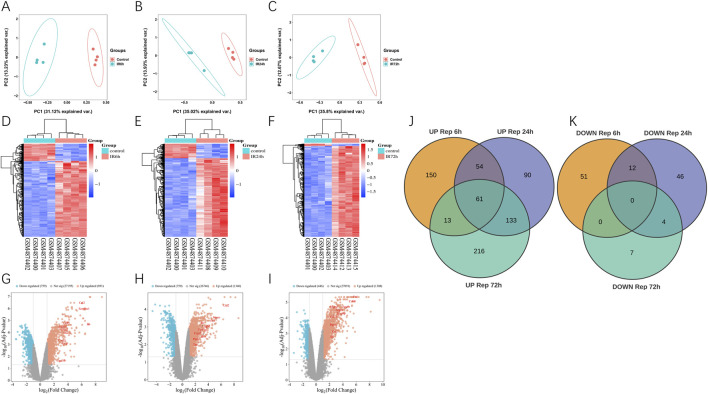
Differentially expression genes analysis in GSE160516. **(A–C)** PCA analysis results. **(D–F)** The heatmap of differentially expression genes identified based on the cutoff criteria of the adjusted P-value <0.05 and |logFC| ≥ 2. The color code depicts the expression of each gene, with blue indicating the lowest and red as the highest expression. **(D)** 6 h post myocardial I/R injury vs. sham. GSM4874400/4874401/4874402/4874403 refers to the myocardial samples from the sham control group, and GSM4874404/4874405/4874406/4874407 refers to the myocardial samples from the myocardial I/R group. **(E)** 24 h post myocardial I/R vs. sham. GSM4874400/4874401/4874402/4874403 refers to the myocardial samples from the sham control group, and GSM4874408/4874409/4874410/4874411 refers to the myocardial samples from the myocardial I/R group. **(F)** 72 h post myocardial I/R vs. sham. GSM4874400/4874401/4874402/4874403 refers to the myocardial samples from the sham control group, and GSM4874412/4874413/4874414/4874415 refers to the myocardial samples from the myocardial I/R group. **(G–I)** The volcano plot of differentially expression genes identified based on the cutoff criteria of the adjusted P-value <0.05 and |logFC| ≥ 2 at 6, 24, 72 h post myocardial I/R. The red dots represent upregulated DEGs and the blue dots indicate downregulated DEGs. **(J, K)** Venn diagram presenting the DEGs between myocardial I/R and sham control samples at 6, 24 and 72 h post-reperfusion. A total of 61 upregulated DEGs were common and no overlapping differentially expression genes that were downregulated among all three post-reperfusion time-points. DEGs, differentially expressed genes; I/R, ischemia/reperfusion.

We used GEO2R to find differentially expressed genes (DEGs). After 6 h of reperfusion, 341 DEGs were found (278 gene expression increased and 63 decreased); 399 DEGs were observed at 24 h of reperfusion (337 upregulated and 62 downregulated); and 431 DEGs were detected at 72 h of reperfusion (420 upregulated and 11 downregulated). Principal component analysis showed differences of samples between the ischemia-reperfusion 6, 24 and 72 h groups (Figures 1A–C). Heat maps and volcanic maps visually demonstrated these results (Figures 1D–I). The Venn diagram showed the DEGs that were common among ischemia-reperfusion groups (Figures 1J,K; Table 2). 61 upregulated common DEGs were found in multiple groups, but no downregulated common DEGs were found in multiple groups.

**TABLE 2:** Overlapped DEGs between Con-IR6h, Con-IR24h, and Con-IR72 h microarray datasets. (DEGs, differentially expressed genes).

DEGs	Genes
Upregulated	CTGF, THBS1, CCL2, TIMP1, MYC, CXCL5, IL1RN, SELP, HMOX1, PRG4, MNDA, MS4A6D, IL6, IL4RA, CD44, HP, RAI14, VCAN, TGFBI, ITGAM, LILRB4A, ADAM8, CCR1, CLEC4D, CD53, LOX, GLIPR1, PDPN, B4GALT5, FCGR3, TMEM173, LILR4B, MIR7050, ANXA2, FCGR2B, EMB, GPR35, SERPINA3N, PLEK, AA467197, MSR1, CCL9, MIR7676-1, ATP8B4, EMP1, BNC2, TNC, ADAM12, CSF3R, SIRPB1A, CCL3, SAMSN1, CD300LF, TLR13, SLFN4, DPEP2, MMP14, MS4A4C, CD80, CHIL3, CCL12

### Functional enrichment analysis

**FIGURE 2:**
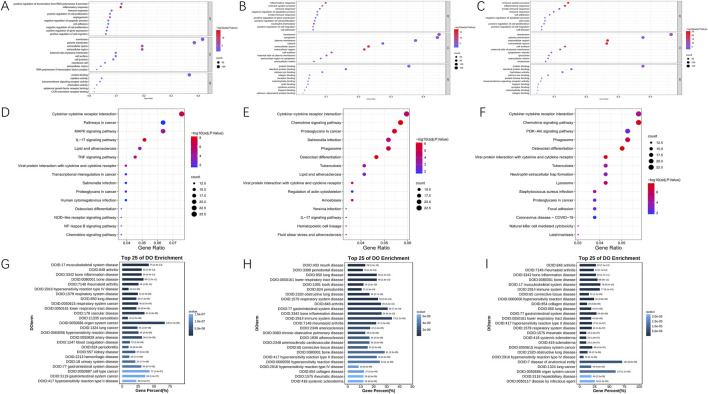
Functional annotation of DEGs in 6, 24 and 72 h post-reperfusion via GO, KEGG, and DO enrichment analyses. **(A–C)** The Gene Ontology (GO) annotations for biological process of the 10 most significant GO enrichment terms. The vertical axis represents enriched GO categories and the horizontal axis represents the ratio of enriched genes in the selected category to all genes analyzed in the GO enrichment analyses. **(A)** Dot plot shows the differentially expressed genes (DEGs) at 6 h post-reperfusion; **(B)** Dot plot shows the DEGs at 24 h post-reperfusion; **(C)** Dot plot shows the DEGs at 72 h post-reperfusion. **(D–F)** The Kyoto Encyclopedia of Genes and Genomes (KEGG) pathway analysis of the 15 most significant KEGG enrichment terms in 6, 24 and 72 h post-reperfusion. The vertical axis represents enriched KEGG categories, whereas the horizontal axis represents the ratio of enriched genes in the selected category to all genes analyzed in the KEGG enrichment analyses. **(D)** Dot plot shows the differentially expressed genes (DEGs) at 6 h post-reperfusion; **(E)** Dot plot shows the DEGs at 24 h post-reperfusion; **(F)** Dot plot shows the DEGs at 72 h post-reperfusion. **(G–I)** Disease ontology (DO) analysis of the top 25 most significant terms in 6, 24 and 72 h post-reperfusion.

To understand the role of DEGs, we used DAVID to analyze their performance in Gene Ontology (BP, CC, MF) classification and the KEGG pathway, including ischemia-reperfusion-6, 24 and 72 h samples and the sham group. The top 10 most prominent GO functions and 15 KEGG pathways of DEGs were shown in Figures 2A–F. Datas showed that Biological Process (BP) items were significantly excessive at all groups, especially those related to inflammatory response and immune response. The enhancement of gene expression was mainly regulated at 6 h and 24 h. The activity of inflammation, immune and innate immune genes were significantly enhanced at 24 and 72 h after reperfusion. Cellular Component (CC) entries related to membrane, plasma membrane, and extracellular space were also very abundant at three time points (6h, 24h, and 72 h). Molecular Function (MF) annotations associated with protein binding was enriched in all reperfusion samples, with cytokine activity and chemokine activity increasing at 6 h, while identical protein binding and calcium ion binding increasing at 24 and 72 h. Cytokine-cytokine receptor interaction and chemokine signaling were significantly enriched in all groups in the KEGG. Furthermore, as shown in Figures 2G–I, the disease ontology (DO) enrichment analysis revealed that most differentially expressed genes (DEGs) in the 6-h reperfusion group were primarily associated with vascular diseases, artery diseases, and inflammatory conditions. In contrast, both the 24-h and 72-h reperfusion groups exhibited a trend toward immune-related disease issues.

### Identification of hub genes within protein interaction networks

**FIGURE 3:**
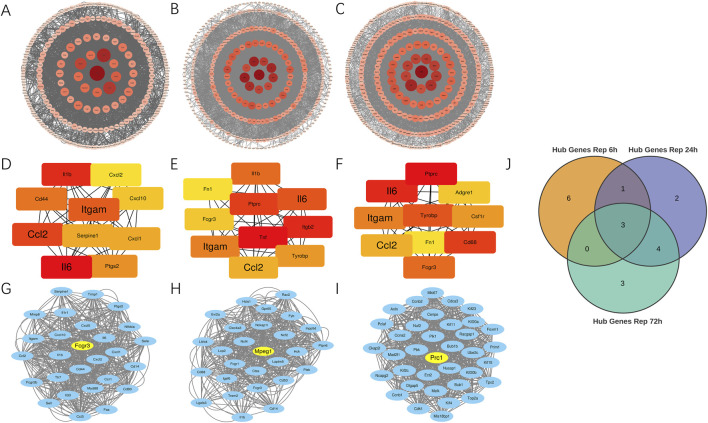
Identification of key hub and seed genes in PPI networks across 6, 24 and 72 h reperfusion groups. **(A–C)** Protein-protein interaction network for DEGs between MIRI and Sham samples at 6, 24, 72 h post-reperfusion. The size of each circle corresponds to a protein’s node degree, with larger circles indicating higher degree. Additionally, the color intensity of the circles reflects this same metric—deeper red hues signify greater node degrees, while lighter shades represent lower degree. DEGs, differentially expressed genes. **(D–F)** Top 10 hub genes at 6, 24, and 72 h post-reperfusion ranked by their degree in the PPI network of DEGs. Each dot represents a hub gene, and the colors of dots indicates the significance of hub genes. Red colors represent relative high degrees of hub genes, while yellow colors represent relative low degrees of hub genes. The lines represent interactions between proteins encoded by these genes. **(G–I)** The Molecular Complex Detection (MCODE) algorithm showed that MCODE 1 contained 27, 27 and 36 gene nodes at 6, 24 and 72 h post-reperfusion. Yellow node represents the Seed gene of each module. **(J)** Venn diagram revealed 3 overlapping upregulated Hub genes (Il6, Ccl2, Itgam).

Using the STRING database, we constructed protein-protein interaction networks across three distinct reperfusion time point groups and visualized them through Cytoscape software (Figures 3A–C). By employing the Degree algorithm via the Cytoscape CytoHubba plugin, we identified the top 10 genes in each reperfusion group’s PPI network as Hub genes, as illustrated in Figures 3D–F. During these three reperfusion periods, Il6, Itgam and Ccl2 were always present in Hub genes. We used Venn plots to show the three genes that were common in different ischemia-reperfusion groups (Figure 3J; Table 3). Other Hub genes included Il1b, Serpine1 and Ptgs2, etc. Through analysis using the MCODE plugin in Cytoscape, we identified three functional modules with the highest scores (21.538, 20.385 and 34.800) in the PPI networks across three reperfusion time point groups. Furthermore, we found that the top 3 genes in these key functional modules—Fcgr3, Mpeg1, and Prc1—were recognized as seed genes, scoring 16.00, 22.58, and 30.00 respectively (Figures 3G–I).

**TABLE 3:** Overlapped Hub genes between Con-IR6h, Con-IR24h, and Con-IR72 h

Overlapping hub genes
ITGAM, CCL2, IL-6

### Constructing miRNAs and TFs-genes regulatory networks

**FIGURE 4:**
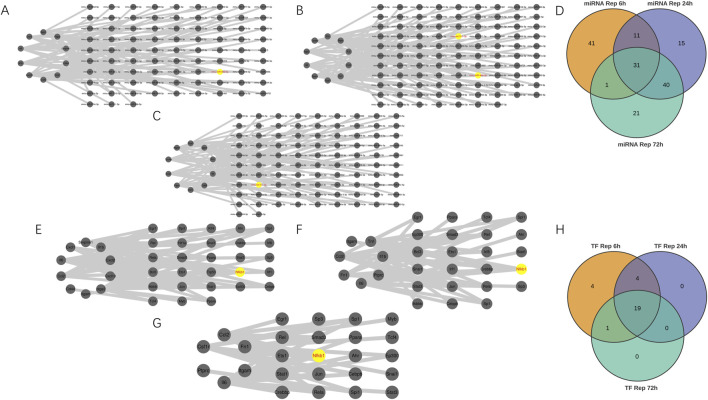
Building miRNA-Hub genes and Transcription Factor-Hub genes Networks. **(A–C)** miRNA–hub genes integrated network. **(D)** Venn diagram revealed 31 overlapping candidate miRNA. **(E–G)** Transcription Factor (TF)-hub genes integrated network. **(H)** Venn diagram revealed 19 overlapping candidate transcription factors.

As shown in Figure 4A, mmu-miR-466L-3p emerged as the dominant microRNA in the 6-h reperfusion group. Network analysis revealed it regulated more hub genes—Cxcl2, Cd44, Ptgs2, Cxcl1, and Il1b—than any other miRNA in this timeframe. Meanwhile, in the 24-h group (Figure 4B), both mmu-let-7c-1-3p and mmu-miR-466L-3p exerted the strongest influence, targeting key genes like Ptprc, Ccl2, Itgb2, and Tnf. The pattern shifted slightly by the 72-h mark (Figure 4C), where mmu-let-7c-1-3p takes the lead, primarily affecting Ptprc and Ccl2. Figure 4D highlighted overlapping miRNAs across ischemia-reperfusion groups, with Table 4 identified 31 miRNAs common to all three time points. We conducted comprehensive investigations into transcription factors (TFs) that may regulate hub genes at different reperfusion time points. Hub genes—PTGS2, TNF, and IL-6—in the three groups were most significantly regulated by 18 TFs (Nfkb1, Jun, Ikbkb, Cebpb, Rela, Sp1, Tcf4, Ppara, Ets1, Ep300, Myb, Klf4, Hif1a, Ahr, Snai1, Pparg, Sp3, Trp53), 13 TFs (Nfkb1, Jun, Ikbkb, Rela, Egr1, Spi1, Sp1, Rel, Bcl3, Irf8, Irf1, Crebbp, Stat3), and 12 TFs (Nfkb1, Jun, Cebpb, Rela, Sp1, Ppara, Ep300, Ahr, Stat1, Crebbp, Stat3, Sp3) respectively. Through comprehensive analysis, it was found that the transcription factor Nfkb1 exerted regulatory effects on the most hub genes across three groups (IR6h, IR24h, and IR72 h), influencing 8 hub genes (Il1b, Cxcl2, Itgam, Cxcl10, Ccl2, Cxcl1, Ptgs2, Il6), 6 hub genes (Il1b, Il6, Fn1, Tnf, Itgam, Ccl2), and 4 hub genes (Ccl2, Fn1, Il6, Itgam) at different reperfusion time points, as shown in Figures 4E–G. The shared transcription factors among these IR groups were identified through observation of Figure 4H. Finally, Table 5 lists 19 co-regulated transcription factors observed across all three groups.

**TABLE 4:** Overlapped miRNAs between Con-IR6h, Con-IR24h, and Con-IR72 h.

Overlapping miRNAs
mmu-miR-493-5p; mmu-miR-466i-5p; mmu-miR-466k; mmu-miR-466L-5p; mmu-miR-466d-5p; mmu-miR-6903-3p; mmu-miR-669c-3p; mmu-miR-6945-3p; mmu-miR-6927-3p; mmu-miR-3473e; mmu-miR-499-3p; mmu-miR-292a-5p; mmu-miR-290a-5p; mmu-miR-293-5p; mmu-miR-6967-3p; mmu-miR-7681-3p; mmu-miR-5624-3p; mmu-let-7c-1-3p; mmu-miR-7041-5p; mmu-miR-7674-3p; mmu-miR-7030-3p; mmu-miR-6371; mmu-miR-669p-3p; mmu-miR-1961; mmu-let-7c-5p; mmu-let-7a-5p; mmu-let-7f-5p; mmu-let-7b-5p; mmu-let-7j; mmu-let-7i-5p; mmu-miR-98-5p

**TABLE 5:** Overlapped TFs between Con-IR6h, Con-IR24h, and Con-IR72 h microarray datasets. (DEGs, differentially expressed genes).

Overlapping TFs
Sp1; Rel; Nfkb1; Stat1; Jun; Stat3; Sp3; Ahr; Tcf4; Ep300; Snai1; Cebpb; Ppara; Rela; Crebbp; Spi1; Smad3; Egr1; Ets1

### Investigating drug-gene interactions

**FIGURE 5:**
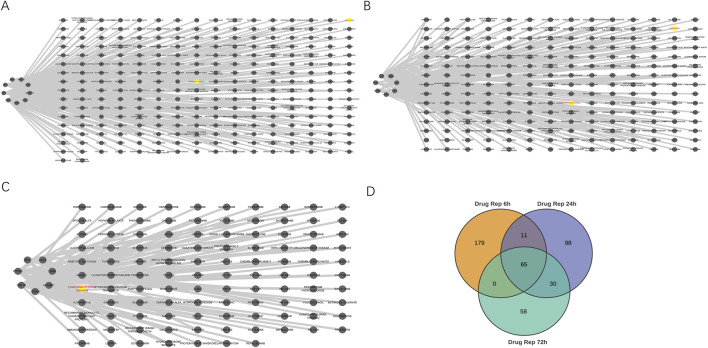
The interaction between drugs and hub genes at different reperfusion time points was analyzed in the GSE160516. **(A–C)** Drugs–hub genes integrated network. **(D)** Venn diagram revealed 65 overlapping candidate Drugs.

We utilized the DGIdb online tool to analyze 441 drugs associated with 19 Hub genes across different reperfusion time point groups, and constructed a drug-gene association map using Cytoscape software (Figures 5A–C). Table 6 presented commonly used drugs in different ischemia-reperfusion (IR) groups, while Figure 5D showed shared drugs among all IR groups. A total of 65 drugs were redundantly used across all three groups. Additionally, EMAPTICAP PEGOL and CARLUMAB demonstrated the highest drug-gene interaction scores (both 17.40) in the 6-h and 24-h reperfusion groups, targeting the critical hub gene Ccl2. In the 72-h reperfusion group, Compound 27 [PMID: 19854648] achieved the highest drug-gene interaction score of 26.10, with Adgre1 as its key drug-target gene. Tables 7–9 listed the top ten drugs ranked by DGIdb interaction scores across the three reperfusion time periods.

**TABLE 6:** Overlapped Drugs between Rep 6h, Rep 24h, and Rep 72 h microarray datasets. (DEGs, differentially expressed genes).

Overlapping drugs
CLARITHROMYCIN; FENTANYL CITRATE; PROTEIN KINASE C INHIBITOR; LIAROZOLE; ATORVASTATIN CALCIUM TRIHYDRATE; PAREGORIC; HYDROCORTISONE BUTYRATE; OXIDOPAMINE; ROVELIZUMAB; PHENYLEPHRINE; THEOPHYLLINE; DIMETHYL SULFOXIDE; PEGINTERFERON ALFA-2B; PEGINTERFERON ALFA-2A; CISPLATIN; ZILTIVEKIMAB; CHINESE HERBS; PF-04236921; MRA 003 US; SIGNAL TRANSDUCTION INHIBITOR; BINETRAKIN; IBUDILAST; INSULIN, REGULAR, HUMAN; SILTUXIMAB; METRONIDAZOLE; CLAZAKIZUMAB; GALLIUM NITRATE; INTERFERON ALFA-2B; DULOXETINE HYDROCHLORIDE; MEDI-5117; GEMFIBROZIL; BIAFINE CREAM; ETANERCEPT; ELSILIMOMAB; SAQUINAVIR; INFLIXIMAB; LINEZOLID; IFOSFAMIDE; OLOKIZUMAB; HMG-COA REDUCTASE INHIBITOR; MIDOSTAURIN; SELENIUM; SIRUKUMAB; ECHINACEA PREPARATION; ENZYME INHIBITOR; FENOFIBRATE MICRONIZED; VITAMIN K; RIBAVIRIN; VITAMIN A PALMITATE; ARSENIC TRIOXIDE; DIHYDROSPHINGOSINE; LEVOFLOXACIN ANHYDROUS; YSIL6; NELFINAVIR; RITUXIMAB; SELECTIVE ESTROGEN RECEPTOR MODULATOR; IMIPENEM-CILASTATIN SODIUM; ADALIMUMAB; VACCINE; EMAPTICAP PEGOL; RECOMBINANT MONOCYTE CHEMOATTRACTANT PROTEIN-1; BINDARIT; RISPERIDONE; CARLUMAB; RS-504393

**TABLE 7:** Top 10 drugs by score ranking in DGIdb at reperfusion 6 h.

Drugs	Regulatory approval	Interaction score	Genes
EMAPTICAP PEGOL	Not approved	17.40126619	CCL2
CARLUMAB	Not approved	17.40126619	CCL2
WWL70	Not approved	13.05094964	CXCL1
WWL123	Not approved	13.05094964	CXCL1
IROPLACT	Not approved	6.14162336	CXCL10
ELDELUMAB	Not approved	6.14162336	CXCL10
NI-0801	Not approved	6.14162336	CXCL10
BINDARIT	Not approved	4.350316547	CCL2
KT-109	Not approved	4.350316547	CXCL1
ABX-1431	Not approved	4.350316547	CXCL1

**TABLE 8:** Top 10 drugs by score ranking in DGIdb at reperfusion 24 h.

Drugs	Regulatory approval	Interaction score	Genes
EMAPTICAP PEGOL	Not approved	17.40126619	CCL2
CARLUMAB	Not approved	17.40126619	CCL2
L19IL2	Not approved	8.031353624	FN1
BINDARIT	Not approved	4.350316547	CCL2
CLAZAKIZUMAB	Not approved	4.261534576	IL6
OLOKIZUMAB	Not approved	4.261534576	IL6
ONFEKAFUSP ALFA	Not approved	4.015676812	FN1
COMPOUND 28 [PMID: 16460935]	Not approved	4.015676812	FN1
L19TNFA	Not approved	4.015676812	FN1
RADRETUMAB	Not approved	4.015676812	FN1

**TABLE 9:** Top 10 drugs by score ranking in DGIdb at reperfusion 72 h.

Drugs	Regulatory approval	Interaction score	Genes
COMPOUND 27 [PMID: 19854648]	Not approved	26.10189928	ADGRE1
EMAPTICAP PEGOL	Not approved	17.40126619	CCL2
CARLUMAB	Not approved	17.40126619	CCL2
L19IL2	Not approved	8.031353624	FN1
BINDARIT	Not approved	4.350316547	CCL2
CLAZAKIZUMAB	Not approved	4.261534576	IL6
OLOKIZUMAB	Not approved	4.261534576	IL6
ONFEKAFUSP ALFA	Not approved	4.015676812	FN1
COMPOUND 28 [PMID: 16460935]	Not approved	4.015676812	FN1
L19TNFA	Not approved	4.015676812	FN1

### Evaluating hub genes diagnostic potential

**FIGURE 6:**
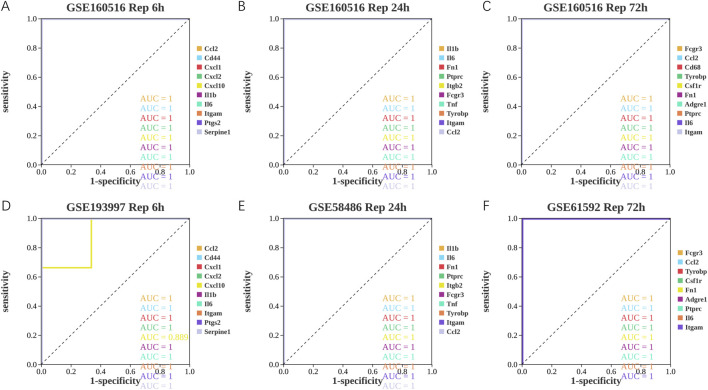
Diagnostic value of hub genes at different reperfusion time points in GSE160516 and external datasets GSE193997, GSE58486 and GSE61592. **(A–C)** ROC curves to assess the diagnostic efficacy of hub genes in GSE160516. **(D–F)** ROC curves to assess the diagnostic efficacy of hub genes in GSE193997, GSE58486 and GSE61592.

As shown in Figure 6; Tables 10–15, for the 6-h reperfusion datasets (GSE160516 and GSE193997), all hub genes, except for Cxcl10 in GSE193997, exhibited perfect diagnostic performance, with an area under the curve (AUC) of 1 (95% CI: 1–1), 100% sensitivity, and 100% specificity. The Cxcl10 gene in GSE193997, however, demonstrated an AUC of 0.889 (95% CI: 0.581–1), with 100% sensitivity, 66.67% specificity, and a cut-off value of 1.02. At the 24-h and 72-h reperfusion time points, all hub genes consistently achieved an AUC of 1 (95% CI: 1–1), along with 100% sensitivity and specificity. These results collectively highlight the high diagnostic accuracy of these biomarkers.

**TABLE 10:** ROC curve analysis, sensitivity, and specificity in GSE160516 at reperfusion 6 h.

	Ccl2	Cd44	Cxcl1	Cxcl2	Cxcl10	Il1b	Il6	Itgam	Ptgs2	Serpine1
AUC (95%CI)	100 (1–1)	100 (1–1)	100 (1–1)	100 (1–1)	100 (1–1)	100 (1–1)	100 (1–1)	100 (1–1)	100 (1–1)	100 (1–1)
Sensitivity (%)	100	100	100	100	100	100	100	100	100	100
Specificity (%)	100	100	100	100	100	100	100	100	100	100
Cut-off value	>8.569	>11.61	>5.579	>5.847	>5.473	>7.287	>6.313	>7.845	>7.845	>10.58

**TABLE 11:** ROC curve analysis, sensitivity, and specificity in GSE193997 at reperfusion 6 h.

	Ccl2	Cd44	Cxcl1	Cxcl2	Cxcl10	Il1b	Il6	Itgam	Ptgs2	Serpine1
AUC (95%CI)	100 (1–1)	100 (1–1)	100 (1–1)	100 (1–1)	88.90 (0.5985–1)	100 (1–1)	100 (1–1)	100 (1–1)	100 (1–1)	100 (1–1)
Sensitivity (%)	100	100	100	100	100	100	100	100	100	100
Specificity (%)	100	100	100	100	66.67	100	100	100	100	100
Cut-off value	>22.61	>5.512	>8.037	>1.590	>5.473	>1.771	>1.609	>3.119	>2.365	>159.9

**TABLE 12:** ROC curve analysis, sensitivity, and specificity in GSE160516 at reperfusion 24 h.

	Il1b	Il6	Fn1	Ptprc	Itgb2	Fcgr3	Tnf	Tyrobp	Itgam	Ccl2
AUC (95%CI)	100 (1–1)	100 (1–1)	100 (1–1)	100 (1–1)	100 (1–1)	100 (1–1)	100 (1–1)	100 (1–1)	100 (1–1)	100 (1–1)
Sensitivity (%)	100	100	100	100	100	100	100	100	100	100
Specificity (%)	100	100	100	100	100	100	100	100	100	100
Cut-off value	>7.104	>4.465	>8.873	>8.912	>5.685	>11.77	>4.064	>8.013	>8.307	>8.352

**TABLE 13:** ROC curve analysis, sensitivity, and specificity in GSE58486 at reperfusion 24 h.

	Il1b	Il6	Fn1	Ptprc	Itgb2	Fcgr3	Tnf	Tyrobp	Itgam	Ccl2
AUC (95%CI)	100 (1–1)	100 (1–1)	100 (1–1)	100 (1–1)	100 (1–1)	100 (1–1)	100 (1–1)	100 (1–1)	100 (1–1)	100 (1–1)
Sensitivity (%)	100	100	100	100	100	100	100	100	100	100
Specificity (%)	100	100	100	100	100	100	100	100	100	100
Cut-off value	>5.272	>4.244	>9.686	>5.959	>6.904	>7.475	>4.747	>6.113	>7.226	>7.027

**TABLE 14:** ROC curve analysis, sensitivity, and specificity in GSE160516 at reperfusion 72 h.

	Fcgr3	Ccl2	Cd68	Tyrobp	Csf1r	Fn1	Adgre1	Ptprc	Il6	Itgam
AUC (95%CI)	100 (1–1)	100 (1–1)	100 (1–1)	100 (1–1)	100 (1–1)	100 (1–1)	100 (1–1)	100 (1–1)	100 (1–1)	100 (1–1)
Sensitivity (%)	100	100	100	100	100	100	100	100	100	100
Specificity (%)	100	100	100	100	100	100	100	100	100	100
Cut-off value	>12.50	>6.603	>12.12	>8.512	>11.30	>9.896	>9.020	>9.378	>4.237	>8.165

**TABLE 15:** ROC curve analysis, sensitivity, and specificity in GSE61592 at reperfusion 72 h.

	Fcgr3	Ccl2	Tyrobp	Csf1r	Fn1	Adgre1	Ptprc	Il6	Itgam
AUC (95%CI)	100 (1–1)	100 (1–1)	100 (1–1)	100 (1–1)	100 (1–1)	100 (1–1)	100 (1–1)	100 (1–1)	100 (1–1)
Sensitivity (%)	100	100	100	100	100	100	100	100	100
Specificity (%)	100	100	100	100	100	100	100	100	100
Cut-off value	>453.0	>366.8	>769.7	>328.2	>911.8	>830.0	>98.13	>97.05	>93.05

### Immune cell infiltration in GSE160516

**FIGURE 7:**
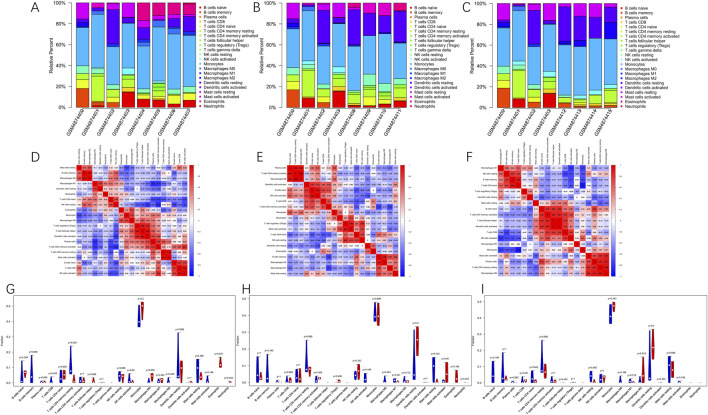
Immune cells infiltration analysis in GSE160516. **(A–C)** The composition of 22 kinds of immune cells in each sample of groups with 6, 24 and 72 h post-reperfusion was shown by histogram. **(D–F)** The correlation between 22 kinds of immune cells in the groups of post-reperfusion at 6, 24 and 72 h was evaluated. Red: positive correlation; blue: negative correlation. **(G–I)** The violin graph shows the difference of immune cells infiltration between MIRI and Sham samples at 6, 24, and 72 h of reperfusion. The Sham samples are shown in blue and MIRI samples are shown in red. The value of p < 0.05 was considered ststistically significant.

A comparative analysis of immune cell populations was performed between MIRI and sham samples at various reperfusion time points in GSE160516. The data presented in Figures 7A–C,G–I revealed significant differences in immune cell distribution across experimental groups. At the 6-h reperfusion time point, a marked decrease in resting CD4 memory T cells (p = 0.029) was observed, alongside an increase in eosinophils (p = 0.021) compared to the sham group. In contrast, the 24-h reperfusion group showed no statistically significant differences in immune cell composition (p > 0.05). The 72-h group, however, exhibited a reduction in the proportion of resting natural killer cells (p = 0.05). Further analysis of immune cell interactions provided additional insights. Correlation matrices evaluating 22 distinct immune cell types revealed dynamic relationships across the time points. Figure 7D illustrates that macrophages and memory B cells exhibited the strongest cooperative interaction at 6 h of reperfusion, while activated CD4 memory T cells and resting NK cells displayed significant antagonism. At 24 h (Figure 7E), synergistic interactions were observed between resting CD4 memory T cells and M0 macrophages, in contrast to the competitive relationship between M1 macrophages and activated CD4 memory T cells. By 72 h (Figure 7F), follicular helper T cells and activated dendritic cells exhibited maximal cooperation, while monocytes and naive CD4 T cells demonstrated the greatest mutual inhibition.

### Validation on independent datasets

**FIGURE 8:**
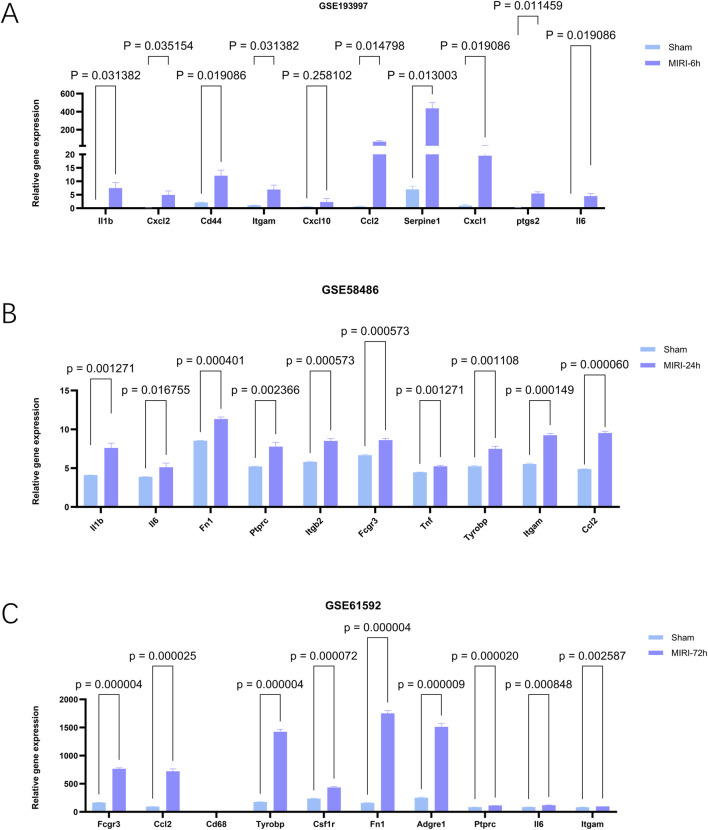
The expression levels of hub genes in mouse myocardial tissues at different reperfusion time points were validated using external datasets GSE193997 (6 h post-reperfusion), GSE58486 (24 h post-reperfusion), and GSE61592 (72 h post-reperfusion). **(A–C)** Expression of genes of interest in external datasets, GSE193997,GSE58486 and GSE61592. The data are expressed as the mean ± standard error of the mean. The value of p < 0.05 was considered ststistically significant.

To investigate the functional roles of hub genes in myocardial tissue following ischemia-reperfusion injury (MIRI), we conducted rigorous analysis across multiple datasets using data processing methods consistent with previous studies to ensure result consistency and reliability. The GSE193997 dataset provided critical expression profiles of hub genes after 6-h reperfusion, with Figure 8A showing that all 10 hub genes (except Cxcl10) exhibited significantly elevated expression levels (p < 0.05). Notably, Serpine1 demonstrated a 400-fold increase in expression post-reperfusion (p = 0.013003), suggesting its potential involvement in ischemia-reperfusion-induced pathophysiological processes. By integrating GSE58486 and GSE61592 datasets, we applied identical analytical methods to examine hub gene expression at 24-and 72-h reperfusion intervals. Data integration enabled clearer visualization of temporal expression patterns. In later analyses, Figure 8B from the GSE58486 dataset revealed sustained elevation of all 10 hub genes’ expression levels (p < 0.05), with Ccl2 showing the most significant increase (p = 0.000060). In the 72-h reperfusion dataset GSE61592, Figure 8C demonstrates that all hub genes showed significantly elevated expression levels (p < 0.05) except for Cd68, which exhibited no notable changes. Notably, Fn1 demonstrated a dramatic 1500-fold increase in expression after reperfusion (p = 0.000004). This study revealed critical alterations in these genes during cardiac ischemia recovery. These findings helped us understand heart damage after ischemia and improve recovery treatment efforts.

### Confirmation of hub genes expression levels and cell viability following oxygen glucose deprivation/reperfusion

**FIGURE 9:**
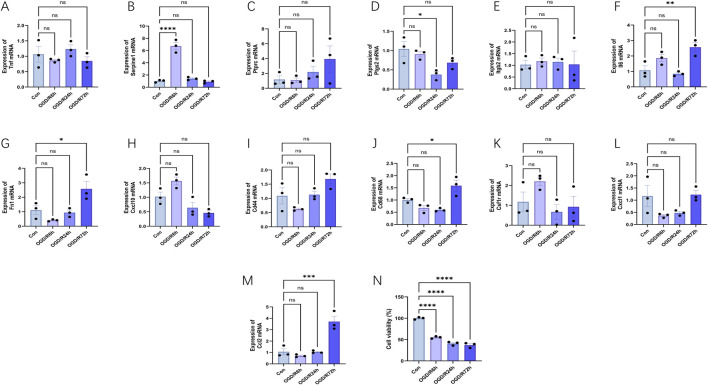
Expression levels of hub genes and cell viability in the oxygen glucose deprivation/reperfusion (OGD/R) model of HL-1 mouse cardiomyocyte lines at different reperfusion time points (6, 24 and 72 h). **(A–M)** HL-1 cells were hypoxic for 6 h and reoxygenated for 6–72 h, and the expression changes of hub genes were verified using RT-PCR (n = 3). **(N)** HL-1 cells were subjected to the OGD process for 6 h and then restored to a normal culture condition for 6 h, 24 h, and 72 h, respectively. And then cell viability was detected with CCK-8 assay. The data are expressed as the mean ± standard error of the mean. *p < 0.05, **p < 0.01, ***p < 0.001, ****p < 0.0001, ns no significance.

Initially, we studied the effect of oxygen glucose deprivation/reperfusion (OGD/R) on cell survival. After HL-1 cells were subjected to the OGD process and then restored to oxygen for 6 h, 24 h, and 72 h, respectively, cell survival decreased significantly (Figure 9N). As expected, the expression levels of most hub genes increased to varying degrees after OGD/R (n = 3) (Figures 9A–M). Within the OGD/R group, there was a notable rise in Serpine1, Ccl2, Il6, Fn1 and Cd68 levels across different reoxygenation stages (p < 0.05), but only Serpine1, Ccl2, Il6, Fn1 were consistent with the verification results in the above external datasets. Notably, specific reoxygenation intervals saw substantial decreases in Ptgs2 levels within the OGD/R group (p < 0.05). Surprisingly, there were no significant differences in the expression of Cd44, Cxcl10, Tnf, Cxcl1, Ptprc, Csf1r and Itgb2. Notably, the Cxcl2, Adgre, Il1b, Fcgr3, Tyrobp, and Itgam expression levels in HL-1 cells were exceptionally low, and the expression levels of these genes could not be detected, so they were not shown in Figure 9.

## Discussion

The myocardial ischemia-reperfusion injury (MIRI) group demonstrated distinct patterns of differentially expressed genes (DEGs) at multiple reperfusion time points compared with the sham group, indicating dynamic transcriptional alterations during ischemia-reperfusion. To elucidate the underlying biological mechanisms and identify key molecular determinants, comprehensive gene expression profiling was performed across these reperfusion intervals. Gene Ontology (GO), Kyoto Encyclopedia of Genes and Genomes (KEGG), and Disease Ontology (DO) enrichment analyses revealed that the DEGs were predominantly enriched in pathways associated with the inflammatory response, immune regulation, cell adhesion, chemokine signaling, and immune- or inflammation-related diseases. These findings underscore that MIRI progression is orchestrated through complex crosstalk between inflammatory and immune-mediated signaling cascades. Protein–protein interaction (PPI) network analysis using the Cytoscape CytoHubba plug-in further identified the top 10 hub genes in each reperfusion group based on the Degree algorithm. Among these, Il6, Itgam, and Ccl2 consistently emerged as hub genes across all reperfusion time points, suggesting that they constitute core components of the molecular framework governing myocardial injury and repair following reperfusion. The integration of miRNA–transcription factor–gene regulatory network analyses revealed that mmu-let-7c-1-3p and mmu-miR-466L-3p miRNAs, together with the transcription factor Nfkb1, regulated multiple hub genes. These regulators may function as pivotal upstream modulators of the inflammatory and immune responses in MIRI, reflecting the layered complexity of transcriptional and post-transcriptional control in cardiac injury. Drug–gene interaction analysis provided additional translational insights, identifying EMAPTICAP PEGOL, CARLUMAB, and Compound 27 (PMID: 19854648) as the top candidates with the highest drug–gene interaction scores across all reperfusion groups. These compounds may represent potential therapeutic agents capable of modulating MIRI-associated signaling pathways. To further validate the bioinformatics predictions, receiver operating characteristic (ROC) curve analyses and immune infiltration assessments were conducted. Moreover, external datasets and an HL-1 mouse cardiomyocyte oxygen-glucose deprivation/reperfusion (OGD/R) model were employed to systematically verify the temporal expression profiles of hub genes. Consistent with computational findings, Serpine1, Ccl2, Il6, and Fn1 exhibited significant upregulation at multiple reperfusion stages (p < 0.05) in both the validation datasets and the OGD/R HL-1 model. Taken together, these results delineate the central role of immune and inflammatory signaling in the pathogenesis of MIRI and identify Serpine1, Ccl2, Il6, and Fn1 as robust biomarkers reflective of distinct reperfusion phases. Their reproducible expression patterns across bioinformatic and experimental models highlight their potential clinical utility in the precision diagnosis, therapeutic targeting, and prevention of myocardial ischemia-reperfusion injury.

Macrophages and T cells are the primary cellular sources of Il6, a cytokine closely linked to the initiation and amplification of inflammatory responses (Shahrivari et al., 2017). Previous studies have demonstrated that Il6 concentrations increase markedly within the first 6 hours following myocardial ischemia-reperfusion injury (MIRI), underscoring its pivotal role in the early inflammatory phase (Iuchi et al., 2018). Moreover, Il6 stimulates diverse cell types to produce and release pro-inflammatory mediators, thereby intensifying local and systemic inflammation (Unver and McAllister, 2018). In patients with myocardial infarction (MI), elevated serum Il6 levels measured on the first day after angioplasty are closely correlated with the subsequent development of heart failure (Hartman et al., 2018). The inflammatory response induced by MIRI is characteristic of the early ischemic and reperfusion stages (Shen et al., 2019; Zhao et al., 2022). Notably, Il6 levels are not only indicative of myocardial injury but also show a strong association with acute myocardial infarction (Frangogiannis, 2021). Beyond pathological conditions, increased Il6 concentrations in healthy individuals have been associated with a heightened risk of cardiovascular complications (Libby, 2021). In patients with acute coronary syndrome (ACS), Il6 serves as a critical biomarker for assessing both the severity of coronary artery disease (CAD) and the risk of mortality (Su et al., 2013; Mowafy et al., 2015; Fanola et al., 2017). Importantly, prior research has shown that inhibition of the Il6 receptor can significantly reduce cardiovascular events, suggesting a promising therapeutic target for the prevention of coronary heart disease (Swerdlow et al., 2012). Furthermore, tocilizumab, a potent Il6 receptor antagonist, has demonstrated robust anti-inflammatory efficacy in patients with non-ST-segment elevation myocardial infarction, as evidenced by substantial reductions in high-sensitivity C-reactive protein (hs-CRP) and cardiac troponin T (cTnT) levels (Kleveland et al., 2016). Collectively, these findings underscore the central role of interleukin-6 in the pathophysiological mechanisms underlying myocardial ischemia-reperfusion injury and highlight its potential as a key therapeutic target.

The Itgam gene, located at p11.2 on chromosome 16, encodes a protein that plays a pivotal role in regulating immune responses by modulating interferon-γ (INF-γ) receptor activity and controlling the production of inflammatory mediators. As a key component of the innate immune defense system (Crispín et al., 2013), Itgam is closely associated with inflammatory processes (Ruparelia et al., 2015). Yue et al. (Yue et al., 2025) reported that during myocardial ischemia followed by reperfusion—the restoration of blood flow after ischemia—Itgam expression in neutrophils and monocytes increased markedly, particularly following the re-establishment of cardiac perfusion. Consistent with these findings, earlier studies demonstrated that Itgam levels are significantly elevated in patients with coronary artery disease (CAD), suggesting its potential diagnostic value for this condition (Liu et al., 2016). Furthermore, experimental evidence indicates that administration of a monoclonal antibody specifically targeting Itgam, an adhesion molecule, can attenuate neutrophil-mediated myocardial ischemia–reperfusion injury (MIRI) in canine models (Simpson et al., 2025). Although the precise molecular mechanism of Itgam in myocardial ischemia-reperfusion injury (MIRI) has not yet been fully elucidated, analysis of external datasets across different time points revealed a significant increase in Itgam expression during MIRI. In contrast, in the oxygen-glucose deprivation/reoxygenation (OGD/R) mouse myocardial cell line HL-1 model, Itgam expression remained unchanged, likely due to its inherently low baseline expression in this cell line. This discrepancy may be attributed to differences in experimental models, as the external datasets were derived from mouse myocardial tissue, whereas the present experiment employed a cell line model. Collectively, these findings suggest that Itgam plays a crucial role in the onset and progression of MIRI and may represent a promising therapeutic target for the prevention and treatment of ischemia-reperfusion injury.

The findings of this study indicate that Ccl2 plays a crucial role at 6, 24, and 72 h after reperfusion. Also known as monocyte chemoattractant protein-1 (MCP-1), CCL2 is essential for regulating the migration and accumulation of various immune cells (Deshmane et al., 2009). Beyond its chemotactic activity, MCP-1 promotes the secretion of cytokines and growth factors, supports tissue regeneration, and contributes to fibrotic remodeling—processes that may facilitate functional recovery following ischemia-reperfusion injury (Kaya et al., 2002; Liu et al., 2015). Nevertheless, sustained or excessive MCP-1 production can provoke chronic inflammation, potentially resulting in prolonged tissue damage (Inoshima et al., 2004; Chao et al., 2009; Zhu et al., 2015). These observations suggest that modulating MCP-1 expression could provide a potential therapeutic avenue for mitigating myocardial ischemia-reperfusion injury.

Differentially expressed gene (DEG) analysis revealed significant enrichment in the cytokine–receptor interaction and chemokine signaling pathways, with members of the chemokine and cytokine families predominating among key genes identified at 6, 24, and 72 h after reperfusion. These findings align with established evidence that reperfusion-induced inflammatory responses markedly enhance chemokine production (Frangogiannis, 2007). Following myocardial ischemia, chemokine expression is regulated by activation of the toll-like receptor (TLR) pathway, reactive oxygen species (ROS) generation, and the NF-κB signaling cascade, with reperfusion further amplifying their transcriptional activity (Jiang et al., 2020). Among specific genes, Ccl2 emerged as a central component of the protein–protein interaction network across all reperfusion phases (6, 24, and 72 h), corroborating previous findings that identified it as a critical mediator in post-ischemic inflammatory regulation (Tarzami et al., 2002). *In vivo* investigations have similarly reported elevated Ccl2 expression following myocardial reperfusion (Chandrasekar et al., 2025). Functionally, inhibition of CCL2 has been shown to mitigate post-ischemic left ventricular remodeling by prolonging the inflammatory phase, delaying macrophage infiltration, and attenuating macrophage-mediated effects, collectively reducing myocardial damage. Other chemokines, notably Cxcl1 and Cxcl2, belong to the CXC chemokine subfamily and are primarily produced by neutrophils infiltrating ischemic myocardium during reperfusion injury. These molecules promote leukocyte recruitment and cellular activation, thereby intensifying the inflammatory milieu (Dewald et al., 2004). Similarly, Cxcl10 expression increases markedly after ischemia and reperfusion in both murine and canine models, although its mRNA levels decline by 24 h post-reperfusion (Liberale et al., 2021), suggesting dynamic temporal regulation during the recovery process. Turning to cytokines, interleukin-1 (IL-1) has been extensively characterized as a pivotal mediator in the inflammatory phase of myocardial ischemia–reperfusion injury, contributing to both pathological and reparative processes. Clinical studies further indicate that patients with acute coronary syndrome treated with IL-1 receptor antagonists exhibit reduced systemic inflammation and lower circulating inflammatory markers (Morton et al., 2015; Suzuki et al., 2025), thereby conferring myocardial protection against ischemia–reperfusion–induced injury.

Upstream regulators of the hub genes were identified using miRDB for miRNA–target gene prediction and TRRUST for transcription factor–target gene prediction. Among these regulators, mmu-let-7c-1-3p, mmu-miR-466L-3p, and the transcription factor Nfkb1 were found to modulate the largest number of hub genes across all three reperfusion time points. Previous studies have provided insights into the biological roles of these regulators. Chi et al. reported that let-7c facilitates the infiltration of immune cells into ischemic and underperfused tissues, while simultaneously activating multiple endothelial repair pathways (Chi et al., 2020). Consistently, Ha Sen Ta Na et al. observed that elevated let-7c expression confers protection against ischemia and promotes functional recovery following spinal cord ischemia/reperfusion injury (Ha et al., 2019). In contrast, the functional involvement of miR-466L-3p in myocardial ischemia-reperfusion injury (MIRI) remains largely undefined, despite its known associations with inflammation, immune regulation, tumorigenesis, and neural differentiation (Li et al., 2012; Ward, 2013; Yu et al., 2017; Zhang et al., 2020). Furthermore, the transcription factor Nfkb1 has been extensively implicated in orchestrating the inflammatory response during MIRI (Wang et al., 2020). The current findings demonstrate that miR-let-7c-1-3p, mmu-miR-466L-3p, and Nfkb1 collectively regulate the majority of hub genes within the constructed network. This convergence underscores their potential significance as central regulatory elements and highlights them as promising directions for future research into the molecular mechanisms underlying MIRI.

To assess the diagnostic potential of the identified hub genes across different reperfusion time points in myocardial ischemia-reperfusion injury (MIRI), receiver operating characteristic (ROC) curve analysis was performed using dataset GSE160516, followed by validation in three independent external datasets (GSE193997, GSE58486, and GSE61592). In the 6-h reperfusion dataset (GSE193997), Cxcl10 exhibited a moderate diagnostic performance with an AUC of 0.889. In contrast, all other hub genes in the remaining datasets demonstrated perfect discrimination, achieving an AUC of 1.0. Although established biomarkers such as B-type natriuretic peptide (BNP), N-terminal pro–B-type natriuretic peptide (NT-proBNP), cardiac troponin (cTnT), and creatine kinase are routinely employed for the diagnosis of acute myocardial infarction (AMI) due to their high sensitivity and specificity, they may not adequately reflect the pathophysiological processes underlying MIRI. Consequently, the lack of reliable biomarkers specific to MIRI continues to pose a major diagnostic challenge. In this context, the present findings provide valuable insights and a potential reference framework for the identification of novel and specific biomarkers capable of improving the early detection and clinical evaluation of MIRI.

We analyzed immune cell infiltration in myocardial ischemia-reperfusion injury (MIRI) using the CIBERSORT algorithm and further assessed the correlations among immune cell populations in GSE160516. The analysis revealed significant associations between several immune cell subtypes and MIRI. Specifically, eosinophil levels increased in the 6-h reperfusion group, whereas resting CD4 memory T cells and resting natural killer (NK) cells decreased in both the 6-h and 72-h reperfusion groups. Moreover, key hub genes—Serpine1, Il6, Ccl2, and Fn1—showed strong correlations with immune cell infiltration, emphasizing the close interplay between hub gene expression and immune system activity. These findings are consistent with prior studies demonstrating the critical role of the innate immune system in the progression of cardiovascular diseases (Swirski and Nahrendorf, 2018). Following myocardial ischemia-reperfusion, immune cells infiltrate the myocardium and initiate a robust inflammatory cascade that compromises cardiac function and elevates mortality risk (Carbone et al., 2020). During the subsequent repair phase, persistent low-grade inflammation driven by immune cell activation may further exacerbate myocardial remodeling, thereby contributing to the onset and progression of heart failure. Indeed, elevated levels of immune cells have been frequently observed in patients with heart failure (Yndestad et al., 2009; Sharma et al., 2023). Collectively, these results suggest that the identified hub genes associated with immune cell infiltration may represent promising therapeutic targets for myocardial ischemia-reperfusion injury. Nonetheless, additional studies are required to elucidate the precise mechanistic relationships between these hub genes and immune cell dynamics in MIRI.

This study explored the association between specific genes and myocardial ischemia-reperfusion injury (MIRI), revealing distinct changes in gene expression within HL-1 cells subjected to different reperfusion conditions. These findings may guide the development of novel therapeutic strategies for cardiac ischemia-reperfusion injury. Despite these advances, several limitations should be acknowledged. Although hub genes were successfully identified and critical time points for their expression changes were determined, further validation through *in vivo* experiments is necessary to clarify their precise biological roles. Moreover, while this study characterized alterations in key genes across different reperfusion states, the specific biological functions and underlying regulatory mechanisms remain to be fully elucidated. Addressing these aspects in future research will be essential to translate these molecular insights into clinically relevant applications.

## Conclusion

Il6, Itgam, and Ccl2 were identified as hub genes across the three reperfusion groups. Bioinformatics analysis indicated that these genes are key factors at distinct stages of myocardial ischemia-reperfusion injury (MIRI). Investigating these genes enhances our understanding of the biological pathways and molecular mechanisms underlying cardiac injury. Analysis of HL-1 cell line gene expression and external datasets revealed significant differences in Serpine1, Ccl2, Il6, and Fn1 expression. These findings indicate that these genes may influence future MIRI therapies and support further research into potential therapeutic strategies.

## Data Availability

The datasets presented in this study can be found in online repositories. The names of the repository/repositories and accession number(s) can be found below: https://www.ncbi.nlm.nih.gov/geo/, GSE160516.
